# Perceptions of Scientific Authorship Revisited: Country Differences and the Impact of Perceived Publication Pressure

**DOI:** 10.1007/s11948-021-00356-z

**Published:** 2022-02-23

**Authors:** David Johann

**Affiliations:** 1 ETH Library, ETH Zurich, Rämistrasse 101, 8092 Zurich, Switzerland; 2 Institute of Sociology, University of Zurich, Andreasstrasse 15, 8050 Zurich, Switzerland

**Keywords:** Scientific authorship, Authorship perceptions, Pressure to publish, Science studies, Germany, Austria, Switzerland

## Abstract

Relying on data collected by the Zurich Survey of Academics (ZSoA), a unique representative online survey among academics in Germany, Austria, and Switzerland (DACH region), this paper replicates Johann and Mayer's (Minerva 57(2):175–196, 2019) analysis of researchers' perceptions of scientific authorship and expands their scope. The primary goals of the study at hand are to learn more about (a) country differences in perceptions of scientific authorship, as well as (b) the influence of perceived publication pressure on authorship perceptions. The results indicate that academics in Switzerland interpret scientific authorship more leniently than their colleagues in Germany and Austria. The findings further indicate that, as perceived pressure to publish increases, researchers are more likely to belong to a group of academics who hold the view that any type of contribution/task justifies co-authorship, including even those contributions/tasks that do not justify co-authorship according to most authorship guidelines. In summary, the present study suggests that action is required to harmonize regulations for scientific authorship and to improve the research culture.

## Introduction

Being named as an author or co-author of a scientific publication traditionally fulfills several functions: It enables readers to recognize who has done the work and who is responsible for it, but also who is entitled to receive credit and reputation based on the publications (e.g., Albert and Wager, 2003; Birnholtz, 2006; Johann & Mayer, 2019; Johann, Rathmann, et al., 2021; Teixeira da Silva & Dobránszki, 2016). Consequently, being mentioned as (co-)author of a scientific publication requires researchers to have made a significant contribution to the publication, following most guidelines and recommendations on scientific authorship (e.g., Hess et al., 2015; Hesselmann et al., 2021; Johann & Mayer, 2019; Osborne & Holland, 2009).

Admittedly, what exactly constitutes a significant contribution is not always clearly spelled out (Czesnick, 2020; Hess et al., 2015; Johann & Mayer, 2019; Swiss Academies of Arts & Sciences, 2013; Whetstone and Moulaison‐Sandy 2020). Some guidelines define tasks that qualify for co-authorship as the participation in study design, data analysis, interpretation of data, or writing of the text (British Sociological Association, 2001; German Research Foundation, 2013; International Committee of Medical Journal Editors n.d.; for an overview of major journals’ authorship criteria, see Hesselmann et al., 2021; Johann & Mayer, 2019). While some guidelines require each co-author to have contributed to several or even all of these tasks and not just to one (e.g., British Sociological Association, 2001; German Research Foundation, 2013), other activities are usually considered as insufficient for co-authorship. These include obtaining or providing funding for research or supervising doctoral students (Austrian Agency for Research Integrity, 2019; British Sociological Association, 2001; German Research Foundation, 2013, 2019; Hess et al., 2015; Hesselmann et al., 2021; International Committee of Medical Journal Editors n.d.; Johann & Mayer, 2019; Osborne & Holland, 2009; Swiss Academies of Arts & Sciences, 2013).

Against this background, a number of studies have been published that address the meaning and practices of scholarly authorship (e.g., Hesselmann et al., 2021; Jabbehdari & Walsh, 2017; Johann & Mayer, 2019; Marušić et al., 2011; Osborne & Holland, 2009; Smith & Williams-Jones, 2012; Wren et al., 2007). Recent articles suggest that a significant proportion of scientific publications contain co-authors who have not contributed adequately to the final manuscript (e.g., Dotson et al., 2011; Jabbehdari & Walsh, 2017; Koepsell, 2017; Wren et al., 2007). For example, relying on a survey among promotion committee representatives at medical schools accredited by the Association of American Medical Colleges, Wren et al. (2007) found that about 40 percent consider it common practice for authorship to be awarded to researchers who do not meet journal authorship criteria.

The aforementioned findings of Wren et al. (2007) fit well with a study by Johann and Mayer (2019). Drawing on survey data from the German Center for Higher Education Research and Science Studies (DZHW), collected in 2016, Johann and Mayer examined the extent to which researchers' perceptions of scientific authorship differ from authorship regulations. They show that a majority of researchers in Germany (over 55 percent) hold perceptions of authorship that do not correspond with the definition of authorship provided by the German Research Foundation (DFG), the most important funder of research in Germany. Johann and Mayer also identified some differences in perceptions of authorship across scientific disciplines. For example, their results suggest that researchers in the natural sciences and medical and health sciences exhibit a wider understanding of authorship than their colleagues in the humanities and social sciences (Johann & Mayer, 2019).^1^

The findings of Wren et al. (2007) as well as Johann and Mayer (2019) are not surprising, given that researchers may face a “prisoner’s dilemma” (Shaw, 2014): Ideally, researchers should follow the authorship guidelines that apply to them and they should not attach too much importance to their publication record and the impact^2^ of their publications. However, they may fear the potential consequences when following this ethical code of conduct strictly, knowing that other researchers who do focus heavily on their publication record and impact of their publications, and who interpret authorship more leniently, may gain a competitive advantage over them, e.g., better chances to acquire third-party funding or to advance their careers (Shaw, 2014; see also Rivera, 2018).

Kovacs (2017) also points out that it might be difficult to adhere fully to authorship guidelines, such as those of the International Committee of Medical Journal Editors (ICMJE), because research funding is allocated on the basis of research results, which, in turn, are often measured by the quantity and quality of publications. Kovacs argues that principal investigators may not be able to afford not having their names included in papers produced with the funding they acquired, even though they may not have contributed significantly to the relevant papers; if they adhered to the authorship guidelines and did not claim honorary authorship, their chances of raising the next third-party funds would decrease (Kovacs, 2017).

Using new data collected by the Zurich Survey of Academics (ZSoA, Rauhut et al., 2021a, 2021b), the research at hand replicates Johann and Mayer's (2019) analysis of researchers' perceptions of scientific authorship and expands their scope: Firstly, the present study is not limited to Germany, but also includes Austria and Switzerland. Secondly, it also examines the role that the perceived pressure to publish plays in researchers' perceptions of scientific authorship. The study at hand thus provides deeper insights into the question why some researchers internalized perceptions of authorship that do not correspond with the current guidelines on authorship in the different countries. At the same time, this research contributes to understanding where to start improving regulations on authorship, thus helping to make suggestions how to avoid authorship disputes. As such, the study at hand follows Smith and Williams-Jones' (2012) suggestion to explore and compare practices of authorship in various scientific disciplines, since, as Smith and Williams-Jones state, such studies both help to deal with tensions and conflicts that arise in different types of research and contribute to the development of commonly accepted procedures for fair attribution of authorship.

The countries of the DACH region are suitable for the present study because, on the one hand, their science systems show relatively strong similarities (Johann, Raabe, et al., 2021; Kreckel & Pasternack, 2008), but, on the other hand, they differ in their policies on scientific authorship and in the degree of perceived publication pressure among researchers, as will be outlined in the next chapter.

## Country Differences and the Impact of Perceived Publication Pressure

The science systems in Germany, Austria, and Switzerland are very similar in some key characteristics (for an overview, see Johann, Raabe, et al., 2021; Kreckel & Pasternack, 2008): For example, performance-based funding has been established in all contexts to make the science systems more competitive. Accordingly, performance criteria in the form of publications and citations play an important role in appointments and promotions. Moreover, due to relative budget cuts, the acquisition of third-party funding—for which the publication record is a central criterion—has gained in importance (e.g., De Boer et al., 2007; Johann, Raabe, et al., 2021; Kehm & Lanzendorf, 2007; Kreckel, 2008; Kreckel & Pasternack, 2008; Orr et al., 2007; Pechar, 2004; Wissenschaftsrat, 2018).

However, in terms of national recommendations for dealing with scientific authorship, the three countries differ significantly:

- The *Guidelines for Good Scientific Practice* by the Austrian Agency for Research Integrity (2019) emphasize that those people should be named as co-authors who have “made an independent scientific/scholarly contribution or another major contribution” to the publication (p. 9). According to these guidelines, the respective contributions of the co-authors should be identified, if possible. Honorary authorship is explicitly rejected. In addition, (a) cooperating just technically for the purpose of data collection, (b) providing funding and infrastructure to conduct the research, and (c) proofreading of the manuscript are considered as insufficient to warrant co-authorship (Austrian Agency for Research Integrity, 2019).
- The recommendations of the Swiss Academies of Arts and Sciences (2013) on authorship of scientific publications suggest that only those persons should be named as co-authors who have made a substantial contribution to the manuscript. At the same time, the recommendations point out that it is not always easy to define what constitutes a substantial contribution. As in the Austrian guidelines, honorary authorship is explicitly rejected. Apart from that, the recommendations are rather unspecific: Only references to other guidelines are recommended, but without specifically stating which of these should be applied. For example, the recommendations of the Swiss Academies of Arts and Sciences refer to the integrity guidelines of the Swiss Academy of Medical Sciences, which state that (a) a leading position in the research institution and (b) financial and organizational support of the research work alone do not entitle a person to be granted co-authorship (Swiss Academies of Arts & Sciences, 2013; see also Swiss Academy of Medical Sciences, 2002).
- The *Proposals for Safeguarding Good Scientific Practice* of the German Research Foundation (2013) are very specific compared to the guidelines by the Austrian Agency for Research Integrity and the recommendations by the Swiss Academies of Arts and Sciences. In these proposals, it is recommended that only those persons should be named as co-authors of a scientific publication who contributed significantly “[a] to the conception of studies or experiments, [b] to the generation, analysis and interpretation of the data, and [c] to preparing the manuscript, and [d] who have consented to its publication, thereby assuming responsibility for it” (p. 83; see also Johann & Mayer, 2019). The proposals of the German Research Foundation (2013) further explicitly discourage honorary authorships. In addition, various tasks and contributions are listed that do not warrant co-authorship (p. 83): “[a] merely organisational responsibility for obtaining the funds for the research, [b] providing standard investigation material, [c] the training of staff in standard methods, [d] merely technical work on data collection, [e] merely technical support, such as only providing equipment or experimental animals, [f] regularly providing datasets only, [g] only reading the manuscript without substantial contributions to its content, [h] directing an institution or working unit in which the publication originates”.
- In 2019, the German Research Foundation published new *Guidelines for Safeguarding Good Research Practice* (German Research Foundation, 2019). In contrast to their 2013 proposals, the German Research Foundation's new guidelines also explicitly emphasize the authorship of software and data: Authors of scientific publications are defined as people who have “made a genuine, identifiable contribution to the content of a research publication of text, data, or software” (p. 18). The explanatory notes to the guidelines set out what constitutes a “genuine, identifiable contribution”. While according to the 2013 proposals multiple conditions had to be met simultaneously to warrant co-authorship (as indicated by the word “and”; German Research Foundation, 2013, p. 83; see also Johann & Mayer, 2019), under the 2019 guidelines only one of several possible conditions need be met. Authors should have been involved in at least one of the following tasks in a manner relevant to the research (German Research Foundation, 2019, p. 18): “[a] the development and conceptual design of the research project, or [b] the gathering, collection, acquisition or provision of data, software or sources, or [c] the analysis/evaluation or interpretation of data, sources and conclusions drawn from them, or [d] the drafting of the manuscript”. The 2019 guidelines also state that all authors must have agreed to the final version of the publication. Honorary authorship is explicitly rejected. Similarly, under the 2019 guidelines, a leadership position or a supervisory function alone do not entitle an individual to be granted co-authorship (German Research Foundation, 2019).

Summarizing the differences in the guidelines between the three countries (see also Table 1), it can be said that, although the guidelines in Germany are less strict today than they were a few years ago, they are still more explicit and strict than the guidelines in the other two countries. I therefore assume that the likelihood of researchers being stuck in a prisoner's dilemma, as described above, is greater in Germany than in Switzerland, and probably also in Austria. Accordingly, Hypothesis H1 states:

H1: *Academics in Switzerland and in Austria interpret authorship more leniently than their colleagues in Germany*.

It should be noted, however, that researchers may also be guided by the regulations of professional associations and leading journals in their discipline, which are often quite different from the aforementioned recommendations and guidelines, but are usually relatively strict (e.g., Bošnjak & Marušić, 2012; Hesselmann et al., 2021; Johann & Mayer, 2019; see also Teixeira da Silva & Dobránszki, 2016).

**Table 1:** Overview of cross-discipline guidelines/recommendations in Germany, Austria, and Switzerland

	German Research Foundation (2013)	German Research Foundation (2019)	Austrian Agency for Research Integrity (2019)	Swiss Academies of Arts and Sciences (2013)
Who should be listed as co-author?	Authors should have contributed significantly (a) to the conception of the studies or experiments, (b) to the generation, analysis and interpretation of the data, as well as (c) to the preparation of the manuscript	Authors should have been involved in at least one of the following tasks in a manner relevant to the research: (a) development and conception of the research project, (b) gathering, collection, acquisition or provision of data, software, or sources, (c) analysis/evaluation or interpretation of data, sources, and conclusions drawn from them, (d) drafting the manuscript	Authors are those who have made an independent scientific/scholarly or major contribution to the publication	Authors are those who have made a substantial contribution to the manuscript
Who should not be listed as co-author?	Honorary authorship is rejected	Honorary authorship is rejected	Honorary authorship is rejected	Honorary authorship is rejected
	(a) Organizational responsibility for obtaining funding, (b) providing standard research materials, (c) training staff in standard methods, (d) technical work on data collection, (e) technical support (such as providing equipment), (f) providing data sets, (g) reading the manuscript without substantially contributing to its content, and (h) directing an institution or working unit in which the publication was originated alone do not entitle a person to be granted co-authorship	A leadership position or a supervisory function alone do not entitle a person to be granted co-authorship	(a) Cooperating just technically for the purpose of data collection, (b) providing funding and infrastructure to conduct the research, and (c) proofreading of the manuscript are considered as insufficient to warrant co-authorship	(a) A leading position in the research institution and (b) financial and organizational support of the research work alone do not entitle a person to be granted co-authorship

Regarding the perceived pressure to publish, a recent study by Johann, Raabe, et al. (2021), using the same data as the study at hand, suggests that most researchers in the three contexts experience a high level of pressure, with perceived pressure being higher on average in Austria and Switzerland than in Germany. The results by Johann, Raabe, et al. further indicate that perceived publication pressure is unevenly distributed across different groups of academics. For example, perceived publication pressure is relatively high among female researchers, researchers younger than 50, and researchers with high academic status (Johann, Raabe, et al., 2021).

The previous literature also points to the consequences that might accompany high publication pressure: Miller et al. (2011) stress that pressure to publish in peer-reviewed journals, while having undesirable side effects on teaching and creativity in research, may have led to higher research output. Similar arguments can be found in Franzoni et al. (2011) and van Dalen and Henkens (2012); both papers emphasize that stronger competition increases publication performances. In line with this, Fanelli (2012), citing Fronczak et al. (2007), suggests that the average number of articles published by scientists in their career has increased, although the length of careers has decreased—at least in some disciplines. Binswanger (2010) even notes that between 1990 and 2006 the number of scientific publications has grown faster than the global economy and much faster than the production of goods and services in industrialized countries. At first glance, this sounds like an intended effect (van Dalen & Henkens, 2012). However, as Fanelli and Larivière (2016) show, increasing publication pressure is not necessarily associated with greater productivity, but with a larger network and more frequent cooperation on publication projects within and outside the researchers’ institutions.^3^

Admittedly, the increasing complexity of research requires larger teams, and working in teams can have advantages (e.g., Dotson et al., 2011; Fanelli & Larivière, 2016; Johann, Rathmann, et al., 2021; Jones, 2021; Wuchty el al. 2007). For example, a study by Wuchty el al. (2007) indicates that teams produce papers of higher quality which are more likely to be cited in comparison to papers produced by single authors (see also Johann, Rathmann, et al., 2021). However, as Dotson et al. (2011) point out, the increase in the number of co-authors may also have negative consequences in the form that the inherent value of authorship can be extenuated. In other words, a considerable proportion of articles include authors who have not made an appropriate contribution to the final manuscript (e.g., Dotson et al., 2011; Jabbehdari & Walsh, 2017). This is reflected in the high number of so-called gift, guest, or honorary authors (Jabbehdari & Walsh, 2017; see also Hesselmann et al., 2021; Teixeira da Silva & Dobránszki, 2016; Whetstone and Moulaison‐Sandy 2020). In summary, while publication pressure encourages researchers to publish, it can also have unintended negative effects on the researchers’ individual publication practices (e.g., Fanelli, 2020; Hall & Martin, 2019; Hayer et al., 2013). In line with this argument, the present paper posits that scholars who perceive the publication pressure to be high interpret authorship guidelines more leniently, sometimes even neglecting these guidelines completely (see also Albert & Wager, 2003). Accordingly, Hypothesis H2 states:

H2: *The higher the perceived pressure to publish, the more leniently scientific authorship is interpreted.*

## Data and Methods

High-quality data are required to explore country differences in the perceptions of scientific authorship and to investigate how the pressure to publish affects researchers' views of what justifies scholarly authorship. The ZSoA (Rauhut et al., 2021a, 2021b), a large-scale representative online survey of academic and artistic staff at various higher education institutions (HEI) in Germany, Austria, and Switzerland, provides such data (for more information on the ZSoA, see Rauhut et al., 2021b, as well as "Appendix B"). Unlike in Austria and Switzerland, no staff at universities of applied sciences were surveyed in Germany (Rauhut et al., 2021b). To ensure better comparability of the data across the three countries, respondents indicating that they work at universities of applied sciences were excluded from the analysis (Johann, Raabe, et al., 2021).

To measure the researchers' perceptions of scientific authorship, in the ZSoA respondents were presented with a list of ten tasks and asked to indicate whether the corresponding tasks, taken by themselves, justified authorship. This question battery corresponds to the question battery in the DZHW Scientist Survey 2016 (Neufeld & Johann, 2016), on which the analysis of Johann and Mayer (2019) was based. The ten tasks are (1) writing the text, (2) planning the study, (3) processing the data, (4) analyzing the data, (5) acquiring third-party funding, (6) interpreting the data, (7) methodological advice, (8) collection of data or material, (9) leadership role, and (10) doctoral supervision of one of the co-authors (for more information on question wording and coding, see "Appendix B"; see Table 4 (Appendix A), for summary statistics of the ten items). According to Johann and Mayer (2019), the variables were recoded so that 1 indicates that the task is considered as justifying co-authorship, and 0 otherwise.

Following Johann and Mayer (2019), the individual items are included in a latent class analysis (LCA) to determine the respondents' affiliation with groups of academics having very similar views of what justifies scientific authorship.^4^ In order to be able to compare the results, I opt for the 5-class solution, as do Johann and Mayer (2019). The AIC and BIC values (see "Appendix A" Table 5) suggest that a 6-class solution would be preferred. However, it is worth noting that models with more classes generally tend to fit the data better (Geiser, 2011). Moreover, with average latent class membership probabilities for the most likely latent class membership ranging from 0.77 to 0.92 (see "Appendix A" Table 6), the 5-class solution distinguishes between the identified latent classes more clearly in comparison with the 6-class solution.^5^ Thus, the 5-class solution seems to be appropriate from an empirical point of view (for information on how to decide on an adequate solution with a certain number of latent classes, see Geiser, 2011).

I follow a “one-step approach” and estimate the effects of the covariates simultaneously as part of the latent class model, following the recommendation of Bolck et al. (2004) and Linzer and Lewis (2011), who emphasize that this approach leads to less biased parameter estimates. As such, this approach differs from Johann and Mayer (2019), who first determined the latent classes and then estimated a regression model with group affiliation as the dependent variable.

The perceived pressure to publish is measured using a six-point Likert scale. Higher values indicate that the perceived pressure is higher (see "Appendix A", Table 4, for summary statistics; for more information on question wording and coding, see "Appendix B"). For more information on the extent and distribution of perceived pressure to publish in Germany, Austria, and Switzerland, see Johann, Raabe, et al. (2021).

The country in which the respondents work (distinguishing between Germany, Austria, and Switzerland) and the respondents’ perceived pressure to publish serve as the main covariates. Following Johann and Mayer (2019), other covariates include the respondents’ scientific disciplines (distinguishing between Humanities, Social Sciences, Natural Sciences, Life Sciences, and Engineering), as well as their academic status (professor, postdoc, predoc), their categorical age in five groups (< 30, 30 to 39, 40 to 49, 50 to 59, 60 +), and their gender, with women coded as 1 and men as 0 (see "Appendix A", Table 4, for summary statistics; for more information on the question wording and (re)coding, see "Appendix B").

I use the *gsem* package in Stata SE versions 15.1 and 16.0 (StataCorp, College Station, TX, USA) to estimate the latent class regression model (Huber, 2019; MacDonald, 2018).^6^

## Results

Table 2 presents the latent class marginal means and latent class marginal probabilities. The latent class marginal means indicate which tasks are considered sufficient by class members to warrant authorship. The closer the value is to 1, the more likely class members are to perceive the task as sufficient to warrant co-authorship. The latent class marginal probabilities refer to the group sizes of the different classes. About 15 percent of academics belong to the “Writing-Oriented Researchers”. For these scholars, authorship is mostly justified by contributing to the writing of manuscripts. Another 22 percent can be described as “Narrow Definition-Oriented Researchers”, who have a rather confined understanding of authorship, including tasks such as data analysis and interpretation, in addition to producing text. The largest group, approximately 27 percent, classify as “Data-Oriented Researchers” who believe that planning the study, data collection, data processing, data analysis, and data interpretation, in addition to writing text, justify authorship. The remaining two groups are least consistent with current guidelines on scientific authorship: “Stewardship-Oriented Researchers” (15 percent) consider that a leadership role, the supervision of PhD candidates, or even methodological advice justify co-authorship. “Catch All Researchers” (21 percent) tend to view any type of contribution/assignment as sufficient to warrant co-authorship.^7^ The groups of academics I identified correspond with those found by Johann and Mayer (2019). However, the estimated share of groups in the population differs to some extent: Johann and Mayer identified 15 percent of “Writing-Oriented Researchers”, 29 percent of “Narrow Definition-Oriented Researchers”, 33 percent of “Data-Oriented Researchers”, 5 percent of “Stewardship-Oriented Researchers”, and 18 percent of “Catch-Alls” (Johann & Mayer, 2019).

**Table 2:** Latent class marginal means and latent class marginal probabilities

	Writing-oriented researchers	Narrow definition-oriented researchers	Data collection-oriented researchers	Stewardship-oriented researchers	Catch all researchers
Task: Writing text	0.9146	0.9879	0.9472	0.9113	0.9143
Task: Planning the study	0.1575	0.5053	0.7998	0.7051	0.9041
Task: Data processing	0.0009	0.2491	0.8882	0.2572	0.9556
Task: Data analysis	0.0034	0.8646	0.9952	0.7113	0.9930
Task: Acquiring funding	0.0149	0.0952	0.2169	0.3308	0.6484
Task: Data interpretation	0.0912	0.7559	0.8859	0.6792	0.8632
Task: Methodological advice	0.0006	0.0146	0.0900	0.0909	0.1817
Task: Collection of data or material	0.0104	0.0779	0.6264	0.2291	0.7997
Task: Leadership role	0.0199	0.0266	0.0560	0.3366	0.5078
Task: Doctoral supervisor	0.0229	0.0000	0.1140	0.5966	0.7287
Estimated share (marginal probabilities)	14.56%	22.38%	26.98%	15.09%	20.99%

*N* = 12,242. The latent class marginal means indicate which tasks are considered sufficient by the class members to warrant authorship. The closer the value is to 1, the more likely it is that the class members perceive the task as sufficient to justify co-authorship. The latent class marginal probabilities refer to the proportion (group sizes) of the different classes

**Fig. 1:**
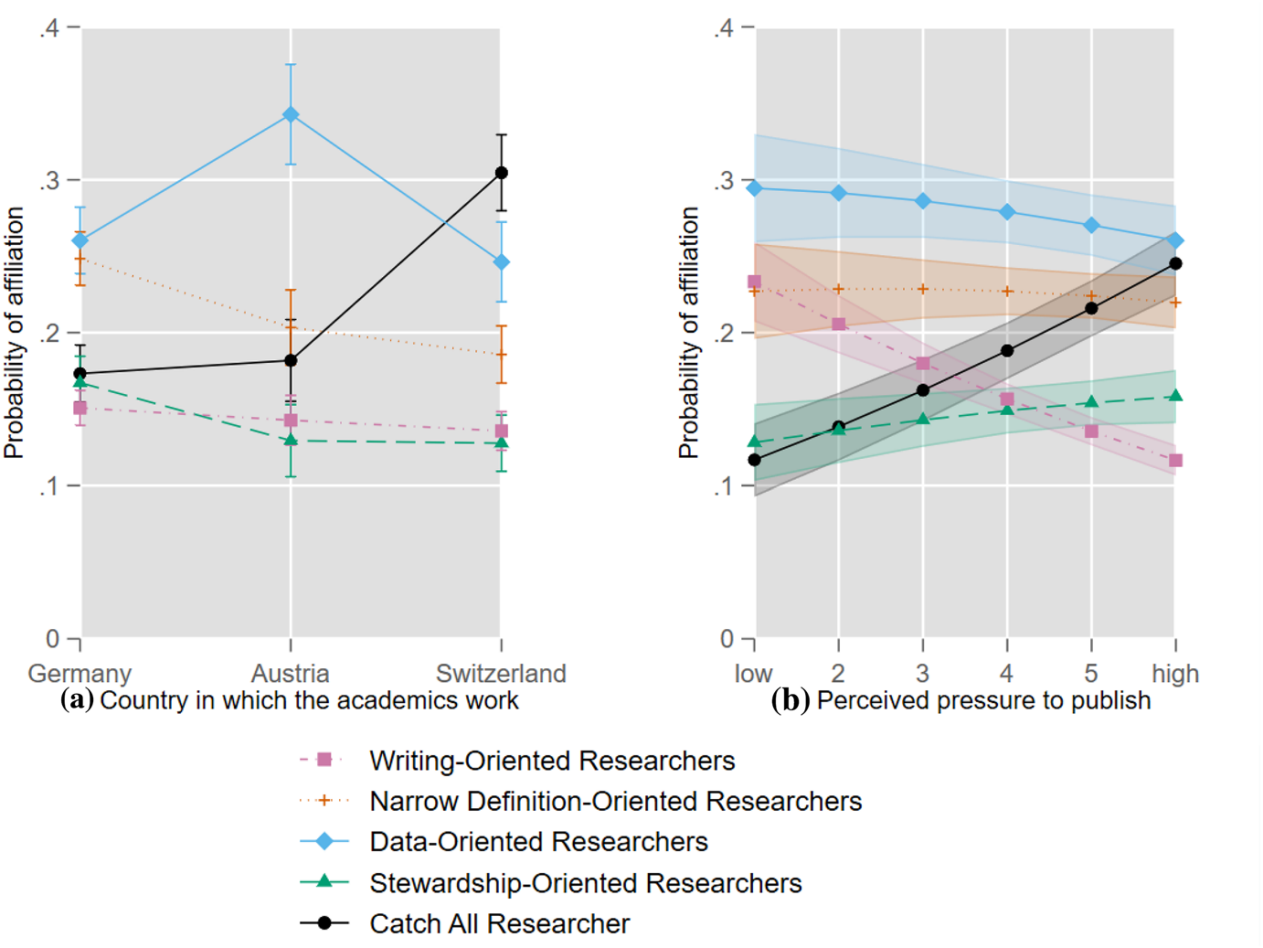
Effects of **a** country and **b** perceived pressure to publish on the probability of affiliation with various types of academics. *Note*: Shown are average adjusted predictions, i.e. predicted probabilities computed according to the “observed-value approach” (Hanmer and Kalkan, 2013; Williams, 2020). Graph produced following Bischof (2017). The symbols (dots, squares, etc.) indicate, for the different values of country and perceived pressure to publish, the estimated probabilities of affiliation with the different types (latent classes) of researchers. The bars/shaded areas indicate the 95% confidence intervals

Turning to the effects of the covariates presented in Table 3, the results indicate that academics in the three countries differ significantly in their perceptions of authorship. Among academics in Germany, the proportion of “Narrow Definition-Oriented Researchers” (25 percent) and “Stewardship-Oriented Researchers” (17 percent) is larger compared to academics in Austria (20 and 13 percent, respectively) and Switzerland (19 percent and 13 percent, respectively). Austrian academics are more likely to belong to the “Data Collection-Oriented Researchers” (34 percent), Swiss scholars to the “Catch All Researchers” (30 percent) (see Fig. 1).

**Table 3:** Average marginal effects of the covariates on the affiliation with various types of academics

	Writing-Oriented Researchers	Narrow Definition-Oriented Researchers	Data Collection-Oriented Researchers	Stewardship-Oriented Researchers	Catch All Researchers
Austria (Ref.: Germany)	−0.0081 (0.0087)	−0.0450** (0.0127)	0.0825*** (0.0159)	−0.0379** (0.0121)	0.0086 (0.0120)
Switzerland (Ref.: Germany)	−0.0151* (0.0073)	−0.0627*** (0.0104)	−0.0140 (0.0128)	−0.0396*** (0.0102)	0.1314*** (0.0107)
Social Sciences (Ref.: Humanities)	−0.1659*** (0.0139)	0.0487* (0.0189)	−0.0465** (0.0157)	0.1412*** (0.0109)	0.0224*** (0.0058)
Natural Sciences (Ref.: Humanities)	−0.3275*** (0.0145)	−0.3214*** (0.0184)	0.1752*** (0.0260)	0.1526*** (0.0140)	0.3212*** (0.0177)
Life Sciences (Ref.: Humanities)	−0.3450*** (0.0145)	−0.3629*** (0.0180)	0.1278*** (0.0304)	0.1429*** (0.0144)	0.4372*** (0.0223)
Engineering (Ref.: Humanities)	−0.2910*** (0.0152)	−0.2950*** (0.0193)	0.0735** (0.0247)	0.2331*** (0.0169)	0.2795*** (0.0179)
Postdoc (Ref.: Predoc)	−0.0066 (0.0091)	−0.0204 (0.0126)	0.0094 (0.0154)	−0.0429*** (0.0122)	0.0605*** (0.0119)
Professor (Ref.: Predoc)	−0.0200 (0.0109)	0.0100 (0.0169)	0.0032 (0.0213)	−0.0467** (0.0174)	0.0536** (0.0178)
30 to 39 (Ref.: < 30)	0.0374*** (0.0088)	0.0175 (0.0140)	0.0072 (0.0166)	−0.0290* (0.0124)	−0.0331* (0.0134)
40 to 49 (Ref.: < 30)	0.0793*** (0.0124)	0.0307 (0.0189)	0.0234 (0.0221)	−0.0511** (0.0172)	−0.0823*** (0.0169)
50 to 59 (Ref.: < 30)	0.1080*** (0.0144)	0.0077 (0.0207)	0.0606* (.0251)	−0.0669** (0.0194)	−0.1094*** (0.0186)
60 + (Ref.: < 30)	0.1064*** (0.0180)	−0.0256 (0.0248)	0.0198 (0.0308)	0.0003 (0.0279)	−0.1003*** (0.0233)
Female (Ref.: Male)	−0.0077 (0.0067)	0.0091 (0.0096)	−0.0433*** (0.0116)	0.0271** (0.0094)	0.0148 (0.0092)
Perceived pressure to publish	−0.0209*** (0.0024)	−0.0031 (0.0036)	−0.0086* (0.0043)	0.0050 (0.0034)	0.0276*** (0.0036)

*N* = 12,242. Standard errors in parentheses; *p < .05, **p < .01, ***p < .001. The average marginal effects can be interpreted as changes in the probabilities of affiliating with the different types of academics. For the categorical covariates, the average marginal effects represent the change from the reference category. Two sample interpretations: (1) researchers from the natural sciences are 32.14 percentage points less likely to be "Narrow Definition-Oriented Researchers" than researchers from the humanities, while (2) researchers aged 50–59 are 10.80 percentage points more likely to be "Writing-Oriented Researchers" than researchers younger than 30

With regard to the effects of the perceived pressure to publish, statistically significant negative effects on the affiliation with the “Writing-Oriented Researchers” and “Data Collection-Oriented Researchers”, as well as a statistically significant positive effect on the affiliation with the “Catch All Researchers”, are observed (see Table 3). These findings suggest that academics perceiving a higher pressure to publish are more likely to belong to the “Catch All Researchers” and less likely to belong to the “Writing-Oriented Researchers” or “Data Collection-Oriented Researchers”. The changes in the predicted probabilities between perceiving high and low pressure are -12 percentage points for the “Writing-Oriented Researchers”, -3 percentage points for “Data Collection-Oriented Researchers”, and + 13 percentage points for the “Catch All Researchers” (see Fig. 1).

The main finding for the other covariates in Table 3 is that large differences in the perceptions of authorship across scientific fields can be observed, which is consistent with the results of Johann and Mayer (2019) (see also Hesselmann et al., 2021). The differences in the predicted probabilities between the scientific fields result in over 30 percentage points in some cases. For example, academics in the natural sciences, life sciences, and engineering are significantly more likely than scholars in the humanities and social sciences to belong to “Catch All Researchers”. Scholars in the humanities and social sciences, on the other hand, are more likely to affiliate with the “Writing-Oriented Researchers” and the “Narrow Definition-Oriented Researchers” than academics in the natural sciences, life sciences, and engineering.

Academic status also seems to play a role in authorship perceptions, even though it appears to be less important than the scientific fields: The results suggest that postdoctoral researchers and professors are more likely to be “Catch All Researchers” and less likely to be “Stewardship-Oriented Researchers” compared to predoctoral researchers. Moreover, younger researchers (those under the age of 30) are more likely to be among the “Catch All Researchers” and “Stewardship-Oriented Researchers” and also less likely to be “Writing-Oriented Researchers” than scholars in other age groups. Finally, women are more likely than men to belong to the “Stewardship-Oriented Researchers” and less likely to affiliate with the “Data Collection-Oriented Researchers”. However, the differences in perceptions between female and male researchers are relatively small, as indicated by the small effect sizes (see Table 3).

## Discussion

This paper replicated and expanded research by Johann and Mayer (2019) on researchers' perceptions of scientific authorship, employing high-quality data collected by the ZSoA. The primary goals of the study at hand were to learn more about (a) country-specific differences in perceptions of scientific authorship and (b) the influence of perceived publication pressure on perceptions of authorship.

One key finding is that researchers are more likely to belong to a group of academics who hold the view that any type of contribution/task justifies co-authorship (even those contributions/tasks that do not justify co-authorship according to most authorship guidelines), as perceived pressure to publish increases. Substantively, this finding is worrying, because it suggests that high publication pressure may tempt researchers to violate the prevailing standards of scientific integrity. This mirrors the results of Hayer et al. (2013) and Hall and Martin (2019), who make a similar argument.

Furthermore, the findings suggest that academics in Switzerland interpret authorship guidelines more leniently than their colleagues in Germany and Austria. At first glance, this finding seems plausible, as the recommendations on authorship of the Swiss Academies of Arts and Sciences (2013) are rather unspecific regarding the tasks that justify co-authorship. At the same time, however, the recommendations of the Swiss Academies of Arts and Sciences emphasize more clearly than many other guidelines that honorary authorship is not permitted (see, for example, Swiss Academies of Arts & Sciences, 2013, p. 5), which raises the question of how seriously the recommendations of the Academies of Arts and Sciences are taken among Swiss researchers.

The study at hand also identified large differences in perception of authorship across disciplines, which is consistent with previous research by Johann and Mayer (2019) and Hesselmann et al. (2021): Scholars in the humanities and social sciences interpret scientific authorship more strictly than their colleagues in other scientific fields. The identified differences in the perceptions of authorship across disciplines may be explained by different tasks that researchers typically perform during the research process, but also by field-specific norms and cultures (Hesselmann et al., 2021; Johann & Mayer, 2019). This is in line with Koepsell (2017), who suggests that authorship of a scientific publication depends on discipline-specific conventions (see also Hesselmann et al., 2021).

The finding that researchers in the three countries and in the different scientific fields differ in their views of what justifies authorship could have undesirable consequences: When researchers from different countries and/or different scientific fields collaborate, which is relatively often the case in the DACH region, particularly due to spatial and linguistic proximity, it is very likely that different ideas about what constitutes scientific authorship will clash. Ultimately, this could lead to disputes about who should or should not be named as the author of a scientific publication (Albert & Wager, 2003; Czesnick, 2020; Johann, Rathmann, et al., 2021; Johann, Velicu, et al., 2020; Smith & Williams-Jones, 2012; Weber, 2018). Future research should therefore address the issue of cross-national and cross-disciplinary collaboration more intensively than has been the case to date and, in this context, examine how the increasing number of co-authors affects attitudes/norms about scientific authorship.^8^

The proportion of “Stewardship-Oriented Researchers” appears to have increased significantly in Germany within four years, comparing the results of Johann and Mayer (2019, data collected in 2016) and the study at hand. This finding insinuates that, at least in Germany, scientific authorship is now interpreted more leniently than a few years ago. Whether this is due to the new, somewhat less strict guidelines published by the German Research Foundation in 2019, or to a shift in perception for other reasons, cannot be determined with the data used here and should be investigated in future research.

The observed age group differences rather support the latter assumption: Younger researchers tend to be more permissive about scientific authorship than their older colleagues, which might indicate that a shift in the perception of authorship among researchers takes place. Among other things, this change in perception might be driven by the fact that the average number of authors per publication has increased (e.g., Johann & Mayer, 2019; Jones, 2021; Rauhut et al., 2018; Wuchty et al., 2007). If this is the case, the results would also imply that Dotson et al. (2011) are correct in claiming that the inherent value of authorship may be extenuated by the increasing numbers of co-authors in scientific publications.

In summary, this study suggests that current national authorship guidelines in the DACH region lack uniformity and that many academics' perceptions of scientific authorship do not match the guidelines in place in their countries. The latter is not surprising, as many researchers face the dilemma of strictly abiding to current authorship guidelines versus career progression (Shaw, 2014). To keep up with the pace of their colleagues and to thrive in their careers, researchers need to publish a lot, and in the most prestigious journals in their field, which is hardly feasible if scholars strictly adhere to current authorship guidelines (Kovacs, 2017; Shaw, 2014). To be fair, however, it should be added that researchers may not be fully aware of the authorship rules that apply to them. It would be desirable for future research to examine how many researchers are aware of the relevant guidelines, and how well they know their content.

What conclusions can be drawn from the present study? *First*, it would be desirable if the regulations on scientific authorship in the three contexts (and beyond) were harmonized in order to set uniform standards. A harmonization of the guidelines appears necessary, as uniform rules for all cooperation partners in international (and interdisciplinary) collaboration may help to avoid disputes over authorship (Albert & Wager, 2003; Czesnick, 2020; Jabbehdari & Walsh, 2017). *Second*, agreement should be reached on the content of the guidelines. In this context, the question arises whether science as a whole is willing to accept more flexible authorship attributions. Given the importance, particularly in disciplines such as the life sciences, of some challenging tasks that actually do not warrant authorship under most current guidelines—such as monitoring of specific techniques or leadership and supervisory roles –, it may be appropriate to redesign the relevant guidelines to acknowledge officially further types of contributions that warrant authorship. A similar view is taken by Clement (2014), who emphasizes that authorship guidelines should include the element of “stewardship”, as senior researchers who wrote the proposal that made the research possible should also be properly credited. However, by expanding the contributions that justify authorship, care should be taken not to dilute the meaning of authorship, otherwise the attribution of authorship may no longer have any value. *Third*, in addition to raising the researchers' awareness of relevant guidelines by clearly communicating the rules and establishing a culture of compliance (Albert & Wager, 2003; Wager, 2009), less emphasis should be placed on publication record in promotion and tenure decisions. *Fourth*, it would be desirable if each publication indicated who made what contribution*.* So-called contribution statements may be beneficial in this context (Johann & Mayer, 2019; Taylor & Thorisson, 2012; Wren et al., 2007). However, in order to avoid a lack of transparency as to how to interpret the given information in the contribution statements, a precise definition and harmonization of contribution statements across disciplines is required, outlining what information needs to be provided, and how (see also Jabbehdari & Walsh, 2017). Another quite similar suggestion comes from Clement (2014), who recommends that journals should publish a matrix according to certain criteria, which, together with some descriptions, shows who contributed what and how much to the paper. Clement also suggests modifying the authorship guidelines to focus on individual responsibilities rather than individual contribution, as it is not only important that authors receive credit for their own contribution, but also that they take responsibility for their own work (Clement, 2014; see also Johann & Mayer, 2019). Given the results in the present paper, this seems to be an appropriate idea that should be pursued. Taken together, the proposed measures may help us to ensure that the average contribution of researchers per publication does not become even smaller than it already is, and that lenient notions of scientific authorship do not become an unofficial standard.

## Appendix A: Additional Tables

See Tables 4, 5, 6.

**Table 4:** Summary statistics

	*N*	Mean	Std. Dev	Min	Max
Task: Writing text	12,242	0.9392	0.2389	0	1
Task: Planning the study	12,242	0.6479	0.4776	0	1
Task: Data processing	12,242	0.5349	0.4988	0	1
Task: Data analysis	12,242	0.7782	0.4155	0	1
Task: Acquiring funding	12,242	0.2680	0.4429	0	1
Task: Data interpretation	12,242	0.7051	0.4560	0	1
Task: Methodological advice	12,242	0.0795	0.2705	0	1
Task: Collection of data or material	12,242	0.3904	0.4879	0	1
Task: Leadership role	12,242	0.1813	0.3853	0	1
Task: Doctoral supervisor	12,242	0.2771	0.4476	0	1
Country	12,242	–	–	1	3
Scientific field	12,242	–	–	1	5
Academic status	12,242	–	–	1	3
Age	12,242	–	–	1	5
Gender (female)	12,242	0.4333	0.4955	0	1
Pressure to publish	12,242	4.6679	1.2961	1	6

**Table 5:** LCA model fit statistics

	*N*	AIC	BIC
3-class solution	12,242	108672.4	109117.1
4-class solution	12,242	107023.7	107653.8
5-class solution	12,242	105988.4	106803.8
6-class solution	12,242	105371.9	106372.6

**Table 6:** Average latent class probabilities for most likely latent class membership by latent class

	Writing-oriented researchers	Narrow definition-oriented researchers	Data collection-oriented researchers	Stewardship-oriented researchers	Catch all researchers
Writing-oriented researchers	0.9176	0.0621	0.0001	0.0202	0.0000
Narrow definition-oriented researchers	0.0235	0.8035	0.0963	0.0760	0.0007
Data collection-oriented researchers	0.0000	0.0768	0.7659	0.0544	0.1029
Stewardship-oriented researchers	0.0156	0.0506	0.0796	0.7911	0.0631
Catch all researchers	0.0000	0.0002	0.1177	0.0533	0.8289

For interpretation of the table, see Geiser (2011): The values of the main diagonal are important here; they indicate the average latent class membership probabilities for the class to which the respondents were assigned, and ideally should be about 0.80 or higher. The higher the values of the main diagonal, the more clearly the different classes can be distinguished from each other

## Appendix B: Additional Information

This paper has been written as a part of the project “Social norms, cooperation and conflict in scientific collaborations” (CONCISE). It is part of the author’s research agenda working with the ZSoA to investigate the distribution and consequences of researchers’ perceived pressure. To date, the following papers addressing, among other things, aspects of the wider research agenda on the distribution and consequences of researchers’ perceived pressure have been published or submitted for peer review: Johann, Raabe et al. (2021), Johann, Rathmann, et al. (2021), and Kessler et al. (2022).

The analysis in the paper at hand is based on the ZSoA (Rauhut et al., 2021a, 2021b). Additional information on the ZSoA (field time, response rate, in which language the questionnaire was completed by how many people) as well as the wording/coding of the variables used are presented below. This information is taken from Rauhut et al. (2021b). Further details, e.g., on the recoding of the variables included in the analysis, are also presented below.

### Field Time and Response Rate

The data collection of the ZSoA took place from 4 February 2020 to 30 April 2020. Up to three invitations were sent to the respondents via e-mail. The overall response rate is approx. 11 percent, with a slightly higher response rate in Switzerland (approx. 14 percent) compared to Germany and Austria (both approx. 10 percent). According to Rauhut et al. (2021b), the response rate of the ZSoA corresponds to the response rates achieved in comparable studies, e.g., the surveys conducted by the DZHW (Ambrasat et al., 2020; Neufeld & Johann, 2016).

### Case Numbers by Country

After cleaning the data, i.e., dropping the cases that have missing values and excluding respondents indicating that they work at universities of applied sciences, the number of cases is *N* = 12,242 (Germany: *N* = 6,794, Austria: *N* = 2,070, Switzerland: *N* = 3,378).

### Perception of Authorship

To measure the researchers’ perceptions of scientific authorship, a question battery of ten items was used. Respondents were asked: “Depending on the discipline or institution, authorship and acknowledgements are handled differently. In publications you are involved in, which of the activities or functions mentioned below justifies, on its own, naming the person as a co-author, and which merit a mention in the acknowledgements? The person was solely… involved in writing the text [Item 1], involved in planning the study on which the text is based [Item 2], involved in processing the data [Item 3], involved in analysing the data [Item 4], involved in the acquisition of third-party funding [Item 5], involved in interpreting the data [Item 6], advising on the application of particular methods [Item 7], involved in the collection of data or material [Item 8], in a leadership role (without any practical or content-related contribution) [Item 9], the doctoral supervisor of one of the co-authors [Item 10].”

While the wording of the German-language version of this question battery from the ZSoA corresponds to the wording of the question battery utilized by Johann and Mayer (2019), which originates from the Scientist Survey of the DZHW (Neufeld & Johann, 2016), the English translation of the question battery differs slightly in both studies. However, the majority of ZSoA respondents filled out the German-language version of the questionnaire, while only a minority (17.5 percent) resorted to the English-language version, which is why differences in translation should not have a large effect on the results. Approx. 7 percent of the respondents filled out the French-language version of the questionnaire.

Respondents had the option to answer the questions with “mention as author”, “mention in the acknowledgements”, and “neither”. Following Johann and Mayer (2019), for analyses, the response options “mention in the acknowledgements” and “neither” were combined and dichotomous variables derived, which were coded as 1 if the activity/function is considered sufficient to be named as a co-author and coded as 0 if the activity/function is considered insufficient to be named as a co-author.

### Perceived Pressure to Publish

The perceived pressure to publish was measured using a six-point Likert scale ranging from “Don’t agree at all” (1) to “Agree completely” (6). Respondents were asked “How much do you agree with the following statements about your working conditions in academia? In my subject area, there is considerable pressure to publish”.^9^ For more information on the extent and distribution of perceived pressure to publish in Germany, Austria, and Switzerland, see Johann, Raabe, et al. (2021).

### Academic Status

With regard to academic status, a distinction was made between predoctoral researchers, postdoctoral researchers, and professors. This variable (“status2”) was created by the ZSoA survey team and provided with the data. Two questions in particular were used by the survey team to measure academic status and build the variable. The wording of these questions was: (1) “Please tell us your current employment position. If you are already retired or have emeritus status, please indicate this.” (2) “Have you completed a doctorate?”.

### Discipline

In the ZSoA, subject areas were surveyed using a categorical variable with 19 response options. For the analysis, the subject areas were grouped into five fields/disciplines (Humanities, Social Sciences, Natural Sciences, Life Sciences, and Engineering), following the DFG subject classification system (*DFG-Fachsystematik*^10^). Deviating from the DFG subject classification, however, a distinction is made between Humanities and the Social Sciences, since significant differences between the two scientific fields/disciplines were suspected with regard to perceptions of authorship. This approach corresponds with Hesselmann et al. (2021).

## Footnotes

1 Similar differences in perceptions of scientific authorship between researchers from different disciplines are found by Hesselmann et al. (2021), whose analysis is based on the DZHW Scientist Survey conducted in 2019/2020. For more information on the DZHW Scientist Survey 2019/2020, see Ambrasat et al. (2020).

2 “Impact” refers to how often scientific publications are cited and is considered an indicator of the importance of scientific publications (e.g., Garfield 2003; Lankhorst and Franchignoni 2001; Larivière and Costas 2016; see also https://guides.library.illinois.edu/c.php?g=621441&p=4328608 (accessed 24 June 2021)).

3 The study by Fanelli and Larivière is based on publication patterns of more than 40.000 researchers looking at the average number of co-authors per paper and considering fractional and first-author publication rates. In order to determine the fractional research productivity of a researcher, the total number of papers published is divided by the average number of (co-)authors listed on their papers (Fanelli and Larivière 2016; see also Fanelli 2020).

4 LCA is a statistical method that can be used to classify respondents into homogeneous subgroups (e.g., Geiser 2011; Linzer and Lewis 2011). LCA has been used in various social science contexts, e.g., to identify political participant types (e.g., Johann, Steinbrecher, et al., 2020; Oser 2010, 2017) or groups that differ in their patterns of use of various substances (Göbel et al., 2016).

5 For the 6-class solution, the average latent class membership probability for the most likely latent class membership is less than 0.75 for two groups.

6 For the data analysis in Stata SE, I also use several user-written commands/packages: the *estout*, *eststo*, *estpost*, and *esttab* commands (from the *st0085_2* package) by Ben Jann (Jann, 2007a; see also http://repec.sowi.unibe.ch/stata/estout/, accessed on 25 January 2022), the *fre* command by Ben Jann (Jann, 2007b), the *gr0070* package by Daniel Bischof which includes new graphic schemes for Stata (Bischof, 2017), and the *grc1leg* command by Vince Wiggins. For complex latent class models, Geiser (2011) recommends replicating the models using other statistical software. Following this recommendation, the latent class regression model is also estimated using Mplus version 8.7 (Muthén & Muthén, 1998–2021). I use the textbook by Geiser (2011) as a guide for conducting LCA in Mplus. The analyses with Mplus version 8.7 and in Stata SE using *gsem* yield the same results.

7 If covariates are omitted, the same pattern of latent classes can be identified.

8 This suggestion fits well with the objectives of the project in the context of which this paper was written (see Acknowledgements), but also with the objectives of the project “PHOENIX—Autorschaft im Wandel” [“PHOENIX—Authorship in Transition”] (https://www.wihoforschung.de/wihoforschung/de/bmbf-projektfoerderung/foerderlinien/qualitaetsentwicklungen-in-der-wissenschaft/qualitaetsentwicklungen-in-der-wissenschaft-i/phoenix/phoenix.html; accessed on 8 October 2021), funded by the German Federal Ministry of Education and Research (BMBF). Furthermore, workshops such as “Benefits and challenges of collaboration in research”, organized by the DZHW and Heinrich Heine University Düsseldorf, seem to be a step in the right direction in this context.
